# Anxiety among patients undergoing various dental procedures

**DOI:** 10.6026/97320630018982

**Published:** 2022-10-31

**Authors:** Siraj DAA Khan, Faisal Ali Alalhareth, Hadi Faraj Moshabab Alyami, Mohammad Abdulkareem Ahmed Alnaji, Abdullelah Abdulkareem Al Touk, Saleh Saeed Saleh Alyami

**Affiliations:** 1 Department of Preventive Dental Sciences, Faculty of Dentistry, Najran University, KSA

**Keywords:** Dental anxiety, anxious, MDAS

## Abstract

Dental Anxiety (DA) was the most frequent problem which can lead to the avoidance of treatment. Therefore, is to evaluate the DA level among patients of different treatments. A questionnaire was distributed among the participants. The Modified Corah Dental Anxiety Scale (MDAS) was used to measure dental anxiety. In gender differences, it has been found that females were more anxious than male participants. The younger age group (18-30 years) was less anxious than the older age groups (P < 0.05). Most of the participants in all conditions were slightly anxious. Male participants exhibited less dental anxiety than females. This might be due to males being more stable emotionally.

## Background:

The common condition which is associated with surgical and medical risks and avoidance of dental treatment is Dental anxiety. In a stressful condition, as a physiological response, it can increase blood pressure and heart rate, dizziness, excessive sweating, pallor, dizziness and flight response. [1] DA is also one of the major factors that impair dental treatment, thus providing a challenge to professional care. [2,3] Filling, root canal therapy, subgingival scaling, extraction, deep probing and other invasive procedures were the most reported procedures associated with pain in patients who have dental anxiety. [4] In a study which involves a group of patients receiving dental hygiene maintenance, those who had issues of dental anxiety anticipated more pain from different procedures i.e. scaling, probing and vibrating sensations. [5] However, the literature indicates that regular dental visits can reduce dental fear. [6] People who experienced dental anxiety or fear are likely to avoid or delay visits to the dentist and a large number of patients with fear fail to show up for appointments or cancel them. [7] Also the patients with dental fear both children and adults are difficult to treat, showed behavioural problems and require more time which leads to unpleasant and stressful conditions for patients as well as for dentists. [8] Finally, these dentally anxious people, due to their avoidance, often have poor dental health. [9] Therefore, it is of interest to evaluate the anxiety level among patients who were undergoing different restorative and endodontic procedures.

## Material and Methods:

After getting an ethical clearance from Scientific research Ethical Committee (442-40-44056 DS) a questionnaire was distributed among 795 patients who were having any type of restorative and endodontic treatment at different dental centers of Najran. The questionnaire had two parts. The 1st part contained the questions about demographic details including the experience of a past dental visit. The second part had questions about different situations related to dental treatment that can cause anxiety. The aim of the study was clearly explained to the participants. Descriptive statistics in terms of frequency and percentage were used to analyze the compiled data and for correlation chi-square test was used with SPSS.

## Results:

A total of 795 people participated in this survey. 564 were female and 231 were male. 571 were aged between 18-30 years, 186 were 31-40 years old and 38 participants were aged more than 40 years. Most of the people (569) had bachelor-level education. Only 4 participants were not educated. In context with their nationality, only 11 persons were non-Saudi. A large number of participants (758) had an experience of a past dental visit. Out of those 758 participants, 390 had an excellent while 32 had a bad experience (Figure 1-see PDF). Among female participants, 220(27.7%) were slightly anxious when they have a dental visit while most of the male participants 131(16.5%) were not anxious. 215(27.0%) female participants said they will feel slightly anxious while sitting in the waiting room for dental treatment and 124(15.6%) male participants were not anxious. About the question of tooth drill, 190(23.9%) were slightly anxious and 40 (5.0%) were extremely anxious while 115(14.5%) males were not anxious and only 1(0.1%) participant responded extremely anxious. 135 (17.0%) and 187(23.5%) female participants were not anxious and slightly anxious when they will have tooth restoration. Most of the female participants 155(19.5%) were fairly anxious while most of the male participants 94(11.8%) were not anxious about the local anaesthetic injection. All responses to this comparison showed significant (P<0.01) relation (Table 1-see PDF). When the response of patients was observed in comparison with their age, the result showed that 219 (27.5%) participants 18-30 years old were not anxious when they have a dentist visit and 11 (1.4%) participants of the same age group were extremely anxious. Among the group more than 40 years old 9 (1.1%) were not anxious, 16(2.0%) were fairly anxious and 1(0.1%) were extremely anxious about their dental visit. While sitting in the waiting room for dental treatment, 188 (23.6%), 80 (10.1%) and 14 (1.8%) from the age group of 18-30 years, 30-40 and more than 40 years were fairly anxious respectively. Most of the participants were from every age group, 190 from 18-30 years. 65 from 30-40 years and 11 from more than 40 years said that they will feel slightly anxious about tooth drill. About the question of restoration and polishing of teeth, 208(26.2%) people aged between 18-30 years said they will not feel anxious while 23(2.9%) were extremely anxious. 44(5.5%) participants said that they will feel not anxious among the age group of 30-40 years and 5(0.6%) from more than 40 years responded in the same way. A large number of participants 205(25.8%) from all age groups agreed on being slightly anxious when they have the local anaesthetic injection in their gums (Table: 2-see PDF). All responses to this comparison were significant (P<0.05). A total of 795 people participated in this survey. About the dental visit, the percentage of participants who were slightly anxious was the highest (35.8%) and the lowest percentage for those who were extremely anxious (2.1%). While sitting in the waiting room the highest percentage of participants (37.1%) were not anxious and 2.8% were extremely anxious. Most of the participants (33.5%) were slightly anxious when they have tooth drill, followed by the not anxious (27.7%), fairly anxious (22.0%), very anxious (11.7%) and extremely anxious (5.2%).The same trend was found in the response about restoration and polishing of teeth. The percentage for slightly anxious, not anxious, fairly anxious, very anxious and extremely anxious was 33.0, 32.3, 18.5, 11.9 and 4.3 respectively. The majority of the participants (25.8%) were slightly anxious about LA injection while only 10.7% were extremely anxious (Figure 2-see PDF).

## Discussion:

In the present study, the comparison between male and female participants showed that females experienced more Dental Anxiety than male participants when they have any kind of dental treatment. The majority of the male was not anxious and only a few were extremely anxious. Same-gender differences were also reported in previous reports on dental anxiety that in comparison to males, females had more fear of the dentist. [10]These results were in accordance with another finding in which females showed higher mean MDAS scores. [11] This could be due to that females have less tolerance to pain and lower pain threshold. [12] For the treatment of tooth drilling and LA injection, high levels of anxiety were reported in females. [11] Same response was reported in our study. The current study showed that the young people (18-30 years old) were less anxious than the other age groups. Participants who were more than 40 years old were more anxious. These results could be due to the past experience of participants. In contrast to our findings, several studies reported an inverse relationship between dental anxiety and age. [12] Lower DA was found in older people. [11]Higher means for MDAS consistent were reported in younger age groups [13], and an increase in anxiety was found in early years of age. This may be due to increased exposure over time allowing patients to develop a tolerance to treatment, and therefore have less anxiety as they age.

## Conclusion:

 Overall it has been concluded from the results that in all conditions most of the participants were slightly anxious and only a few patients were extremely anxious. However, female patients exhibited more dental anxiety than males and people of the young age were more anxious than other age groups.

## Payment/services info:

All authors have declared that no financial support was received from any organization for the submitted work.

## Financial relationships:

All authors have declared that they have no financial relationships at present or within the previous three years with any organizations that might have an interest in the submitted work.

## Other relationships:

All authors have declared that there are no other relationships or activities that could appear to have influenced the submitted work.

## Readers'comments:

The authors did not cite the following article: Udoye CI et al. J Contemp Dent Pract. 2005 May 15; 6(2):91-8. PMID: 15915208. https://pubmed.ncbi.nlm.nih.gov/15915208/

## References

[1] Facco E (2013). Minerva Anestesiol.

[2] Dantas LP (2017). Med Oral Patol Oral Cir Bucal.

[3] Astramskaite I (2016). Int J Oral Maxillofac Surg.

[4] Maggirias J, Locker D (2002). Community Dent Oral Epidemiol.

[5] Hofer D (2016). BMC Oral Health.

[6] Crego A (2014). Front Public Health.

[7] Armfield JM (2012). Aust Dent J.

[8] Brahm CO (2012). Swed Dent J.

[9] Armfield JM (2009). Community Dent Oral Epidemiol.

[10] Armfield JM (2006). Aust Dent J.

[11] Marie L (2018). BMC Oral Health.

[12] Nayak S (2000). Cross Cult Res.

[13] Thomson WM (2000). Community Dent Oral Epidemiol.

